# Denoising Generalization Performance of Channel Estimation in Multipath Time-Varying OFDM Systems

**DOI:** 10.3390/s23063102

**Published:** 2023-03-14

**Authors:** Yinying Li, Xin Bian, Mingqi Li

**Affiliations:** 1 Shanghai Advanced Research Institute, Chinese Academy of Sciences, Shanghai 201210, China; 2 University of Chinese Academy of Sciences, Beijing 100049, China

**Keywords:** 6G, OFDM, multipath time-varying channel, deep learning, channel estimation, NDR-Net

## Abstract

Although Orthogonal Frequency Division Multiplexing (OFDM) technology is still the key transmission waveform technology in 5G, traditional channel estimation algorithms are no longer sufficient for the high-speed multipath time-varying channels faced by both existing 5G and future 6G. In addition, the existing Deep Learning (DL) based OFDM channel estimators are only applicable to Signal-to-Noise Ratios (SNRs) in a small range, and the estimation performance of the existing algorithms is greatly limited when the channel model or the mobile speed at the receiver does not match. To solve this problem, this paper proposes a novel network model NDR-Net that can be used for channel estimation under unknown noise levels. NDR-Net consists of a Noise Level Estimate subnet (NLE), a Denoising Convolutional Neural Network subnet (DnCNN), and a Residual Learning cascade. Firstly, a rough channel estimation matrix value is obtained using the conventional channel estimation algorithm. Then it is modeled as an image and input to the NLE subnet for noise level estimation to obtain the noise interval. Then it is input to the DnCNN subnet together with the initial noisy channel image for noise reduction to obtain the pure noisy image. Finally, the residual learning is added to obtain the noiseless channel image. The simulation results show that NDR-Net can obtain better estimation results than traditional channel estimation, and it can be well adapted when the SNR, channel model, and movement speed do not match, which indicates its superior engineering practicability.

## 1. Introduction

As a multicarrier modulation technology, OFDM technology has the characteristics of high spectrum utilization and strong anti-multipath transmission ability. It has been widely used in terrestrial radio broadcasting systems such as ATSC3.0 and DVB-T2 and mobile communication systems such as 4G and 5G. In practical wireless environments, signals tend to experience frequency selective fading during transmission due to channel multipath effects. This causes amplitude, frequency and phase distortion of the received signal at the receiver, making it difficult for the receiver to demodulate the correct transmitted signal. Therefore, channel estimation at the receiver is required to obtain accurate channel characteristics. This allows the channel to be compensated accordingly at the receiver to improve the transmission quality and ensure the reliability of the communication. In OFDM systems, the conventional channel estimation method approximates a frequency-selective fading wideband channel as a flat-fading narrowband channel one by one, and channel estimation is performed for each flat-fading channel to obtain a more accurate channel frequency response. This method is suitable in wireless environments where the mobile rate is not high and the Doppler frequency shift is small.

With the rapid development of social and economic, the application of high-speed transportation such as trains and high-speed railways is becoming more and more popular, which greatly shortens people’s travel time. However, wireless channels in high-speed environments are highly time-varying, often accompanied by shifts in the channel model and rapid changes in the Doppler shift. At this point, the narrowband channels in each frequency domain can no longer be approximated as flat, and traditional channel estimation methods such as Least Square (LS) and Minimum Mean Square Error (MMSE) are no longer applicable. Therefore, more sophisticated and targeted measures are needed to estimate the channel. To overcome this problem, data-driven OFDM channel estimation based algorithms have attracted discussions among scholars. Preliminary results show that DL-based OFDM channel estimation algorithms outperform traditional model-driven algorithms in high-speed mobile scenarios. However, the performance of this algorithm depends on the richness of the channel information used. If the designed model deviates significantly from the channel environment, the performance of the algorithm will be much worse compared to the theoretical calculation.

Then how to use the DL-based denoising technique in multipath time-varying systems to further improve the generalization of the algorithm under the environment mismatch while ensuring the accuracy of channel estimation is the focus of this paper. In this paper, we first design a feedforward network $CNN_{N}$ to estimate the noise level of the current environment. Then cascade the DnCNN as a denoising network $CNN_{D}$ to denoise the noisy channel estimation matrix. Finally, use the residual module to improve the convergence speed of the model. The experimental results indicate that the overall implementation complexity of the proposed algorithm is lower than that of the conventional channel estimation algorithm, which improves the engineering practicability of the algorithm.

In general, the main contributions of this paper include:

- This paper uses 5G standard channels and parameters to construct an OFDM communication system, which has excellent engineering practicability.
- A DL-based OFDM channel estimation denoising network NDR-Net is proposed. The receiver first obtains the noisy channel matrix using the traditional channel estimation algorithm. Then it is modeled as a two-dimensional image and fed into a denoising network with $CNN_{N}$ and $CNN_{D}$ cascade. The $CNN_{N}$ is an NLE subnet to extend the generalization noise interval, and the $CNN_{D}$ is a DnCNN with residual layer, which mainly serves to denoise the channel images.
- Through extensive simulations, we verify the performance advantages of the proposed cascade algorithm over traditional algorithms and a single denoising algorithm. Then we evaluate the performance impact of the proposed algorithm trained on fixed SNR, channel model, and velocity, and tested on other SNR, channel model, and velocity points, to verify the adaptability of the proposed algorithm for real time-varying multipath channels.

The remainder of this paper is organized as follows. The related work on DL-based OFDM channel estimation are presented in Section 2. The wireless OFDM communication system model, traditional channel estimation algorithm, and denoising network DnCNN are introduced in Section 3. The proposed denoising channel estimator is illustrated in Section 4. The simulation results are discussed in Section 5. Section 6 concludes this paper.

## 2. Related Work

In 5G communications, traditional signal processing algorithms are inefficient and inflexible in handling large data. It is also difficult to fit the nonlinear operations in the environment. Artificial Intelligence/Machine Learning (AI/ML) technologies are experts in extracting signal features automatically, processing data in milliseconds, and modeling complex wireless environments. Therefore, the convergence of AI/ML technologies with 5G can solve lots of the challenges faced by current channel estimation. In addition, at the latest 3rd Generation Partnership Project (3GPP) meeting, the first projects for the first version of the 5G-Advanced Release 18 (R18) standard were identified, which also includes how AI/ML can be applied to DL-based physical layer transmission technologies.

Many scholars have applied AI/ML techniques to the physical layer of communication, including DL-based autoencoder technology [1,2,3], Modulation recognition technique [4,5], Peak to Average Power Ratio (PAPR) reduction techniques [6,7], Channel State Information (CSI) feedback technology [8,9], Joint source channel coding [10,11]. Therefore, the application of AI/ML techniques to channel estimation has great potential for both improving the accuracy of channel estimation and improving performance in scenarios with diverse channel conditions.

The authors in [12] show the preliminary results of applying deep learning to channel estimation and signal detection, which uses the Fully Connected Deep Neural Network (FC-DNN) to implicitly estimate the CSI and then directly recover the transmitted symbols. The method is experimentally shown to exhibit more superior performance than traditional signal detection methods, but convergence is slow due to too many parameters. To reduce the estimation complexity, the authors in [13] combine the FC-DNN in [12] and the Channel Parameter-Based (CPB) algorithm in [14] to propose the Channel Parameter Refinement Network (CPR-Net). This network can directly estimate the channel parameters and then reconstruct the channel response by channel parameters. The results demonstrate that CPR-Net can significantly improve the estimation accuracy of complex amplitude and Doppler shift, but there is the problem of error propagation. The work in [15] proposed a DL architecture called ComNet. ComNet with a combination of digital and analog drives, using two subnetworks for channel estimation and signal detection, respectively. The results show that the model can achieve higher detection accuracy with significantly reduced parameters compared to the FC-DNN network. The above models consider only time-invariant channels, which are not applicable to the time-varying channels.

Improving the accuracy of channel estimation under time-varying conditions is done in two main ways. One is to find a suitable DL model to estimate the channel response directly as accurately as possible, and the second is to use a suitable denoising algorithm to reduce the noise of the already estimated channel response. For the first method, many scholars have made related studies. The authors in [16] proposed ChanEstNet to address the problem of limited performance of downlink channel estimation due to fast time-varying and non-stationary characteristics in high-speed mobile scenarios. This method first uses CNN to extract the channel characteristic vector and then uses Recurrent Neural Network (RNN) to estimate the channel, so that it learns the features of fast time-varying and non-stationary channels. The work in [17] proposed a novel Internal Carrier Interference Network(ICINet) for sensing Inter-Carrier Interference (ICI). Experiments show that the proposed ICINet can significantly reduce inter-subcarrier Interference and improve the accuracy of existing channel estimation schemes. In [18], the interpolation process in the frequency domain is first simulated using a two-layer CNN. Then the Bi-directional Long Short-Term Memory (BiLSTM) structure is used for channel prediction. Finally, it is input to CNN again to reduce the dimensionality to obtain CSI. The results show that the proposed DL-based algorithm can obtain higher estimation accuracy than the traditional channel estimation algorithm in different environments. The authors in [19] proposed a Linear Machine Learning (LML) based channel estimation and online training scheme. The data are generated in groups by tracking the channel in real-time and a linear regression model is used as the learning module. The results show that the scheme can adapt to channel changes under shorter training cycles and a small amount of training data.

Another way to improve the accuracy of channel estimation is through denoising. The authors in [20] proposed an image noise reduction model Denoising convolutional neural network (DnCNN). The model can learn the noise in the image while cascading on residual learning can accelerate the training process. Simulation results demonstrate that DnCNN has better denoising effect compared with traditional denoising methods and can be extended to handle general image denoising tasks. The authors in [21] consider the millimeter-wave channel matrix as a two-dimensional image and apply a Learned Denoising-based Approximate Message Passing (LDAMP) network, fusing DnCNN to an iterative signal reconstruction algorithm for channel estimation. Simulation results showing that the LDAMP outperforms conventional algorithms and compressed sensing algorithms. The authors in [22] designed a channel estimator based on Knowledge-Driven Machine Learning (KDML) using the DnCNN in large-scale Multiple-Input Multiple-Output(MIMO) systems. The estimator has a simple neural network structure and a low training cost. Simulation results demonstrate that the estimator outperforms the LS algorithm and machine learn-based estimators. The authors in [23] proposed a two-stage channel grid estimation algorithm ChannelNet. Firstly, the time-frequency response of the fast-fading channel is considered as a two-dimensional image, and the Low Resolution (LR) image is obtained by Gaussian interpolation of the pilot value. Then the Super-Resolution Neural Network (SRCNN) algorithm is used to obtain the estimated channel as a High Resolution(HR) image. Finally, the DnCNN is used to remove the noise effect. An improved Convolutional Blind Denoising Network (CBDNet) is proposed as a channel estimator in a mmWave MIMO system [24]. The estimator can estimate the SNR level while reducing the noise in the channel matrix. However, the channel model and the mismatch of moving speed are not considered. According to our current survey, the existing DL-based channel estimation studies mainly stay at the level of improving the generalizability of the model to SNR. And generalizability considering channel model and movement speed has not been available or less studied.

In this section, we comprehensively introduce the application of AI/ML in the direction of channel estimation in OFDM/MIMO communication. Some of these techniques that are used in the high-speed channel and our contribution are summarised and compared in Table 1.

## 3. System Model

In this section, we first introduce the model of the OFDM communication system in Section 3.1. Then introduce the LS algorithm at full pilots in Section 3.2. Finally, we introduce how to reference the DnCNN model to channel estimation denoising in Section 3.3.

### 3.1. OFDM System

**Figure 1:**
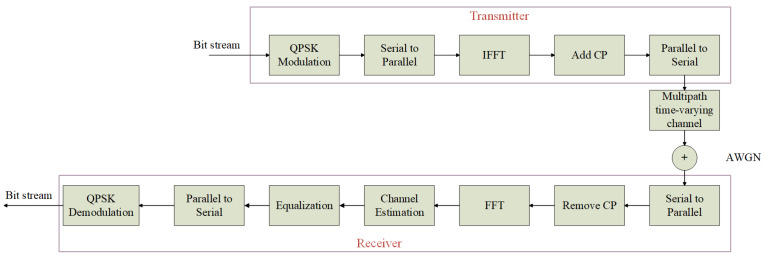
OFDM system architecture for multipath time-varying channels.

Figure 1 shows the block diagram of the OFDM system in the multipath time-varying channel. At the transmitter, each group inputs a bit stream $x=\left\{x_{0},x_{1},x_{2},\cdots ,x_{2N_{c}-1}\right\}$ is digitally modulated to Quadrature Phase Shift Keying (QPSK) symbols X(k), where $N_{c}$ is the number of subcarriers, $x_{i}=\left\{0,1\right\}$, $i=\left\{0,1,2,\cdots ,2N_{c}-1\right\}$. Then the modulated signal is Invert Fast Fourier Transformation (IFFT) to obtain the time-domain signal x(n) of the modulated signal:

(1) $$x\left(n\right)=\mathrm{IFFT}\left[x\left(k\right)\right]=\sum_{k=0}^{N_{IFFT}}X\left(k\right)e^{j\left(\frac{2\pi kn}{N_{IFFT}}\right)},n=0,1,2,\cdots ,N_{IFFT}-1$$

A Cyclic Prefix (CP)of length $N_{g}$ is inserted to eliminate Inter Symbol Interference (ISI) caused by multipath channels, then we can obtain a new OFDM signal $x_{f}\left(n\right)$:

(2) $$x_{f}\left(n\right)=\left\{\begin{array}{cc} x\left(N_{IFFT}+n\right) & ,n=-N_{g},-N_{g}+1,\cdots ,-1\\ x(n) & ,n=0,1,\cdots ,N_{IFFT}-1 \end{array}\right.$$

Considering the multipath time-varying channel of the Single Input Single Output (SISO) model, i.e.,

(3) $$h\left(t\right)=\sum_{l=0}^{L-1}h_{l}\delta \left(t-\tau_{l}\right)e^{j2\pi v_{l}t}$$

where *L* represents the number of resolved multipath, *l* indicates the multipath channel index, $\tau_{l}$ represents the time delay of the *l*-th path, $h_{l}$ denotes the complex amplitude of the *l*-th path, $v_{l}$ denotes the corresponding doppler shift, which can be calculated by the formula $v_{l}=f_{c}v\cdot \cos\theta /c$, $f_{c}$ denotes the carrier frequency, *v* denotes the moving speed of the mobile station, *c* denotes the speed of light, θ denotes the signal arrival angle. Since this paper consider SISO systems, so $\theta =0^{\circ }$, $v_{c}=f_{c}v/c$.

Then the downlink received signal y(n) can be expressed as:

(4) y(n)=x(n)⊗h(n)+w(n)

where ⊗ denotes cyclic convolution, w(n) denotes Additive White Gaussian Noise (AWGN). CP is removed from the received signal, and then through Fast Fourier Transformation (FFT), the following received signal can be obtained:

(5) $$Y\left(k\right)=FFT\left[y\left(n\right)\right]=\frac{1}{N_{IFFT}}\sum_{n=0}^{N_{IFFT}-1}y\left(n\right)e^{-j\left(2\pi kn/N_{IFFT}\right)},k=0,1,\cdots ,N_{IFFT}-1$$

At this time, the received signal Y(k) can also be expressed as:

(6) Y(k)=X(k)H(k)+W(k)

### 3.2. LS Channel Estimation

Since all positions of the OFDM system are considered as pilots in this paper, so we can directly use a simple LS estimate to get ${\hat{H}}_{LS}$, by minimizing the cost function:

(7) $$J\left({\hat{H}}_{LS}\right)={∥Y-X{\hat{H}}_{LS}∥}^{2}=Y^{H}Y-Y^{H}X{\hat{H}}_{LS}-{\hat{H}}_{LS}{}^{H}X^{H}Y+{\hat{H}}_{LS}{}^{H}X^{H}X{\hat{H}}_{LS}$$

Let the partial derivative of the above cost function concerning ${\hat{H}}_{LS}$ be equal to 0:

(8) $$\frac{\partial J\left({\hat{H}}_{LS}\right)}{\partial {\hat{H}}_{LS}}=-2{\left(X^{H}Y\right)}^{H}+2{\left(X^{H}X{\hat{H}}_{LS}\right)}^{H}=0$$

Thus, the solution of LS channel estimation can be obtained as follows:

(9) $${\hat{H}}_{LS}={\left(X^{H}X\right)}^{-1}X^{H}Y=X^{-1}Y$$

The LS algorithm is simple and has low complexity. However, it ignores the effect of noise, which leads to a large error in the estimation. Therefore, denoising is needed after LS estimation.

### 3.3. DnCNN Model

**Figure 2:**
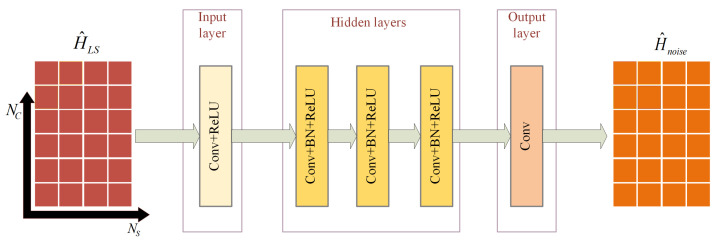
The network structure of the DnCNN.

In this subsection, we model the noisy channel matrix obtained by LS estimation as a two-dimensional image, where $N_{c}$ and $N_{s}$ represent the number of subcarriers and OFDM symbols, respectively. The image is input to DnCNN network for denoising to obtain a noisy image of the same dimension. DnCNN consists of 5 convolutional layers, and its specific structure is shown in Figure 2. The first Conv layer is the input layer and uses 64 different filters of size 3×3×1. The filter is followed by Rectified Linear Unit (ReLU), where ReLU(x)=max(0,x) is a nonlinear activation function. The input layer mainly transforms the input image into an actionable feature map. The hidden layer consists of 3 convolutional layers, each of which uses 64 filters of the size 3×3×1. Each filter is followed by a Batch Normalization (BN) layer and ReLU activation function. BN layer is used because BN can force the input values of each layer neuron back to a standard normal distribution with mean of 0 and variance of 1. That is, the increasingly skewed distribution values after the previous layer of transformation are pulled back to the standard distribution so that the activation input values can fall in the region where the nonlinear function is more sensitive to the input. This prevents the gradient from vanishing and speeds up the convergence. The one-sided suppression of the post-connected ReLU activation function can also be demonstrated, since the action of BN makes the data not distributed on the side of all zeros. The last layer is the output layer, and a 3×3×1 filter is used to reconstruct the noisy image.

${\hat{H}}_{LS}$ is input to the DnCNN to obtain the channel noise image ${\hat{H}}_{Noise}$, so residual learning [25] needs to be added to obtain the channel image $\hat{H}$ after noise reduction. It is expressed as $\hat{H}={\hat{H}}_{LS}-{\hat{H}}_{Noise}$. The reason for choosing DnCNN with residual learning as the denoising module is that through residual learning, DnCNN can implicitly remove potentially clean images using the BN operation, making each input layer Gaussian and reducing correlation. Therefore, BN can be complemented with residual learning to improve image denoising ability. Therefore, BN and residual learning can complement each other to improve image denoising ability.

Although DnCNN has a certain denoising performance under the condition of unknown noise levels, it can only solve the denoising problem under specific noise level. Once the SNR, channel model, or moving speed of the receiver does not match the training model, a large error will be generated. Therefore, it is necessary to know the noise level in the current environment in advance, which can reflect various mismatches, so that DnCNN can be applied to a wider range of noise intervals. However, this is often unknown to the receiver. Therefore, a module is required that is dedicated to estimating the noise level interval of the current environment.

## 4. NDR-Net Assisted Channel Estimation

In this section, we first introduce the architecture and workflow of the proposed channel estimation model NDR-Net in Section 4.1. Then introduce the proposed noise level estimation subnet NLE, and explain the selection of network parameters in Section 4.2. Then introduce the selection of experimental data sets in Section 4.3. Finally, in Section 4.4, we analyze the complexity of the proposed algorithm and compared it with other algorithms.

### 4.1. NDR-Net

**Figure 3:**
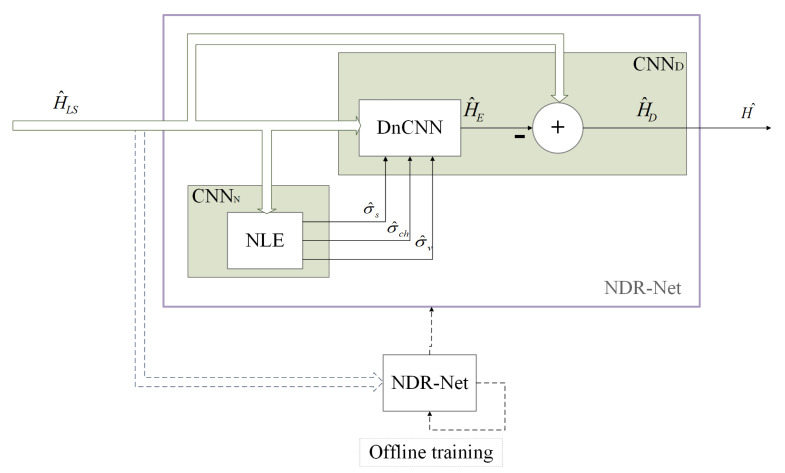
The framework of NDR-Net.

The framework of the proposed NDR-Net based channel estimator is presented in Figure 3. The NDR-Net is composed of noise level estimation subnet $CNN_{N}$ and denoising subnet $CNN_{D}$. The $CNN_{N}$ module is composed of the NLE, and the $CNN_{D}$ module includes DnCNN and Residual learning. The receiver first makes a rough LS estimation of the channel to obtain ${\hat{H}}_{LS}$, then inputs ${\hat{H}}_{LS}$ as the noisy channel matrix to the NLE network to obtain the noise level $\hat{\sigma }$ set. It needs to be noted here that ${\hat{\sigma }}_{s}$ represents the estimated noise level that is the SNR value, ${\hat{\sigma }}_{ch}$ represents the Root Mean Squared (RMS) delay spread corresponding to the estimated channel model, and ${\hat{\sigma }}_{v}$ represents the estimated receiver movement speed. Then inputs $\hat{\sigma }$ set and ${\hat{H}}_{LS}$ together to the DnCNN cascade network to obtain ${\hat{H}}_{E}$, finally use residual learning to obtain ${\hat{H}}_{D}$. It can be expressed as:

(10) $$\hat{H}={\hat{H}}_{D}=f\left(\Theta ;{\hat{H}}_{LS}\right)=f_{D}\left(f_{N}\left(\Theta_{N};{\hat{H}}_{LS}\right);\Theta_{D}\right)$$

where $f_{N}$ and $f_{D}$ represent the functions of $CNN_{N}$ and $CNN_{D}$ models respectively, $\Theta_{N}$ and $\Theta_{D}$ represent the hyperparameters of $CNN_{N}$ and $CNN_{D}$ models respectively. *f* and Θ denote the function and the set of hyperparameters of the NDR-Net model, respectively. The loss function of NDR-Net is expressed as the Mean Square Error (MSE) between the estimated channel response value and the actual channel response value:

(11) $$L=\underset{\theta \in \Theta }{\arg\min}E\left\{∥\hat{H}{-H∥}^{2}\right\}$$

### 4.2. NLE Module

To solve the problem of insufficient performance of DnCNN under unknown noise level, a convolutional and fully connected noise level estimation network is proposed in this paper. The network is used as a feedforward network of DnCNN to achieve adaptive noise by taking the noise level of NLE output as a known condition of DnCNN. When SNR does not match, $\hat{\sigma }$ represents the estimated SNR value of the current environment. When the channel model does not match, $\hat{\sigma }$ represents the root mean square delay spread of the current model. When the moving speed does not match, represents the corresponding Doppler shift value.

**Figure 4:**
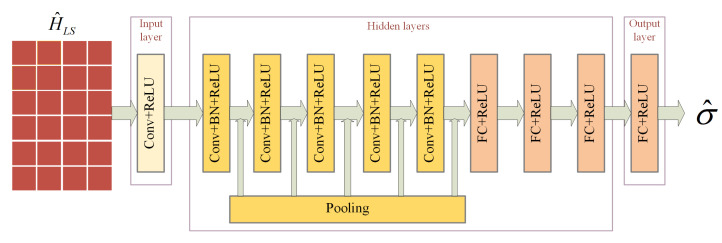
The network structure of the NLE.

The specific structure of NLE is shown in Figure 4, with 10 layers. The first layer is a convolutional layer using 32 different filters of size 3×3×1, followed by a ReLU function. The middle five layers are convolutional layers, which serve the same purpose as the hidden layers of DnCNN, except that the filter size is changed to 32. A pooling layer of size 2×2 is also added after each layer to reduce the network parameters. The last four layers are fully connected layers, which gradually synthesize the features extracted from the previous layer. The number of connection points is 2000, 200, 50, 1, respectively. Each fully connected layer is followed by a ReLU function for nonlinear operations.

#### Selection of Pooling Layers

Pooling includes Maxpooling and Averagepooling. In NLE, in order to determine the final pooling choice, the noisy channel is modeled as an image input to NLE, SNR is used as a label, and NLE is considered as a regression problem for the experiment. SNR was chosen as the label because the changes of channel model and moving speed are based on SNR. Therefore, ensuring the accuracy of SNR estimation is also a prerequisite for improving the generalization performance in other aspects. By employing these two pooling methods separately, the estimates of NLE at different SNRs were recorded. Root Mean Squared Error (RMSE) was used to measure the accuracy of the prediction results. The calculation formula is as follows:

(12) $$\mathrm{RMSE}\left(\sigma \right)=\sqrt{\frac{1}{N}\sum_{i=1}^{N}{\left(\hat{\sigma }-\sigma \right)}^{2}}$$

where *N* indicates the size of the data sample in each SNR group, σ denotes the true SNR value, and $\hat{\sigma }$ denotes the predicted SNR result.Table 2 and Table 3 list the predicted $\hat{\sigma }$ and the corresponding RMSE values for 500 sets of data when σ is equal to 5, 10, 15, 20, 25, 30, 35, respectively. As can be seen from Table 3, the error predicted by using average pooling is smaller than that predicted by maximum pooling at any SNR. The reason for this result is that the network model is not deep in layers, and the use of maximum pooling can easily filter out the useful signals by judging them as noisy signals, which leads to a poorer estimation performance than the average pooling. Therefore, the Averagepooling method is used in the subsequent experiments.

### 4.3. Dataset

In this paper, all data are generated in the same communication system. Firstly, QPSK modulation is used to generate 16,000 sets of guide frequency sequences as the initial data stream. The data stream then passes through the OFDM communication system as shown in Figure 1, and the initial data set is obtained by rough LS estimation of the data at the receiver. Since the network cannot process complex numbers, the real and imaginary parts of each group of data in the data set are constructed in parallel. The reconstructed dataset is split into a training set and a validation set, from which 75% are used for training, 25% are used for validation. Correspondingly, a test set of the same size as the validation set is generated in the same way. For $CNN_{N}$, the labels are composed of SNR, RMS time delay and Doppler frequency shift corresponding to each input dataset (SNR, channel model, and velocity do not match). For the $CNN_{D}$, the labels are the real channel response matrix.

### 4.4. Complexity Analysis

This subsection will represent the complexity of channel estimation algorithms by using the symbol O. For the conventional channel estimation algorithms, the computational complexity required by LS is $O(N_{C})$ and the MMSE algorithm is $O({N_{C}}^{3})$. For AI-based channel estimation algorithms, only the computational complexity of online training needs to be considered because the training process is done in advance and does not occupy real-time computing and storage resources.

For the noise level estimation stage in the NDR-Net algorithm, the $CNN_{N}$ module has a total of L layers, which includes the convolutional part and the fully connected part, and the computational volume is represented by $C_{1}$ and $C_{2}$ respectively. The fully connected layer has 4 layers. For the denoising stage, the $CNN_{D}$ module has a total of H layer, and the computational volume is denoted by $C_{3}$. Then the complexity of NDR-Net can be expressed as:

(13) $$\begin{array}{cc} O\left(C_{1}+C_{2}+C_{3}\right)\; & =O\left(B_{N}E_{N}\left(2N_{c}k_{1}^{2}+n_{L-4}k_{L-4}^{2}+\sum_{l=2}^{L-5}n_{l-1}n_{l}k_{l}^{2}\right)\right.\\ \; & +B_{N}E_{N}\sum_{l=L-3}^{L}n_{l-1}n_{l}\left.+B_{D}E_{D}\left(d_{1}k_{1}^{2}+2N_{c}k_{H}^{2}+\sum_{h=2}^{H-1}d_{h-1}d_{h}k_{h}^{2}\right)\right) \end{array}$$

where $B_{N}$ and $E_{N}$ represent the size of mini-batch and epochs of $CNN_{N}$ module, respectively. $B_{D}$ and $E_{D}$ represent the size of mini-batch and epochs of $CNN_{D}$ module, respectively. $k_{i}^{2}$ denotes the size of kernels in *i*-th layer, $n_{i}$ and $d_{i}$ denotes the number of neurons of $CNN_{N}$ and $CNN_{D}$ at *i*-th layer, respectively. Here for each group of OFDM signals there have $n_{1}=d_{H}=2N_{C}$. As can be seen, the computational complexity of the model is closely related to the number of neurons per layer.

The complexity of other high-speed channel estimation algorithms can also be obtained by the same calculation. The numbers of the parameters and the Floating Point Operations (FLOPs) of these high-speed algorithms are presented in Table 4.

It can be noticed that the computational complexity of NDR-Net is higher than that of ChannelNet and KDML since the $CNN_{N}$ module brings additional computations. The complexity of NDR-Net is lower than that of CBDNet because NDR-Net introduces a pooling module in the network to reduce the computational effort. Since the $CNN_{N}$ module can predict the noise level of the environment in advance, the denoising module of NDR-Net has only 5 layers, which can greatly reduce the training FLOPs. Specifically, although NDR-Net requires 7k more FLOPs than KDML, it can adapt to different physical environments, which is beneficial in general.

It should be emphasized here that due to the time-varying nature of the wireless channels, the specific environmental SNR, channel model, movement speed and data sets have to be taken into account when a network model is trained. The complexity increases linearly with the number of trained models, but this is acceptable in the context of improving the model generalization performance.

## 5. Experimental Evaluation

### 5.1. Parameter Configuration

In this subsection, the generalization performance of the proposed model under different SNR, channel model and velocity will be explored respectively. The simulation is carried out on a computer with Intel (R) Core (TM) i9-9900kf CPU @ 3.60GHz and NVIDIA GeForce GTX 2060 SUPER configuration under 64-bit Windows10 operating system. Among them, the data sets used are randomly generated by 5G channel model on the Matlab simulation platform. The network model is implemented in Keras in Tensorflow. The optimization algorithm is chosen as Adaptive Moment Estimation (Adam).

The main parameters of the communication system used in the simulation are shown in Table 5. Normal CP refers to the 7 OFDM symbols in a time slot, the first OFDM symbol with 160 CP, and the 2–7 OFDM symbols with 144 CP. The movement rate of the simulated multipath channel is 3–30 km/h in TDL-A, 30–120 km/h in TDL-B, and 120–350 km/h in TDL-C unless otherwise specified. The parameters of the proposed network model are shown in Table 6.

### 5.2. Simulation Results

In the following experiments, we will compare the proposed Channel estimator NDR-Net with the traditional LS algorithm, MMSE algorithm [26] and the Channel estimator using only $CNN_{D}$ under different SNR, channel model and velocity. Among them, $CNN_{D}$ denoising algorithm is the basic algorithm. The performance measure is the Channel estimation error MSE, defined as:

(14) $$MSE=\frac{1}{N}\sum_{n=1}^{N}{∥\hat{H}-H∥}_{2}^{2}$$

#### 5.2.1. Generalization of NDR-Net on SNR

In this part, in order to explore the generalization performance of the proposed algorithm NDR-Net on SNR, firstly, the channel model is fixed as TDL-B, and the moving speed is selected as 120 Km/h. In [20], the author has proved that the $CNN_{D}$ trained under a single fixed SNR is not suitable for a large range of SNR environment, so three SNRs are selected to train the $CNN_{D}$ to obtain three denoising models. In this way, after the SNR value is estimated by $CNN_{N}$, a matching denoising model can be selected for channel denoising according to the actual situation, which can ensure that the training complexity is reduced while the estimation performance is improved.

**Figure 5:**
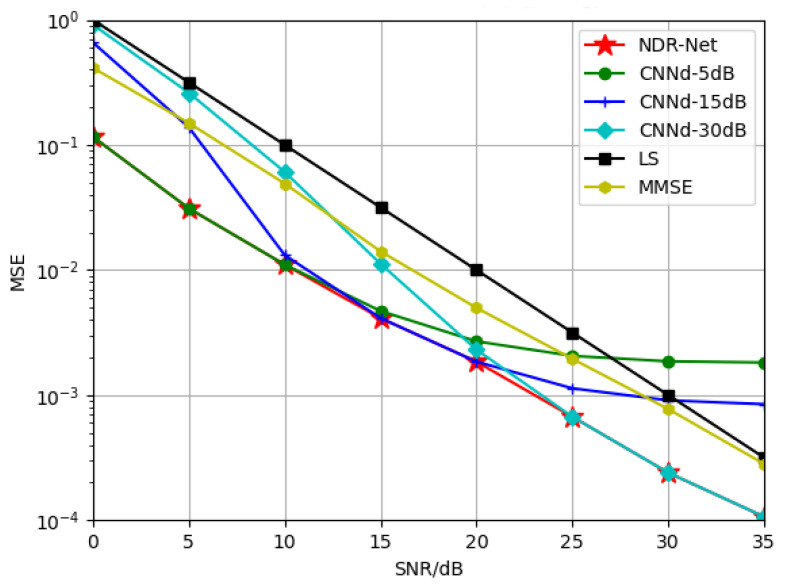
Generalization performance of the trained channel estimator with different SNR for the test channel TDL-B-120Km/h.

Figure 5 compares the generalization performance of SNR by different channel estimation algorithms. As can be seen from the simulation results, the estimation effect of the model obtained by training only $CNN_{D}$ at 5 dB decreases gradually after 15 dB. Due to the existence of virtual subcarriers, the model already appears flat layer at 20 dB, and the estimation effect is lower than that of LS estimation after 20 dB. The serious mismatch of SNR leads to the fact that $CNN_{D}$ has no denoising effect. The $CNN_{D}$ model trained at 15 dB and 30 dB is the same. It can be seen that $CNN_{D}$ can only fit a single SNR and its nearby SNR points, and lacks adaptability to other SNRs, which also proves the conclusion of literature [20] once again. The cascaded network NDR-Net (red line in the figure) can solve this problem well. Because it can adaptively select the best denoising model according to the SNR value in the current actual environment, NDR-Net can achieve good estimation performance on any SNR, and delay the flat layer problem caused by virtual subcarriers. It can be seen that in the 0–35 dB range, NDR-Net can gain 7–10 dB higher than the LS estimation, and 5–7 dB higher than the MMSE estimation.

In addition, we also do generalization performance comparison experiments of these algorithms on SNR under fixed other channel models and moving speeds, and the generalization results are similar.

#### 5.2.2. Generalization of NDR-Net on Velocity

**Figure 6:**
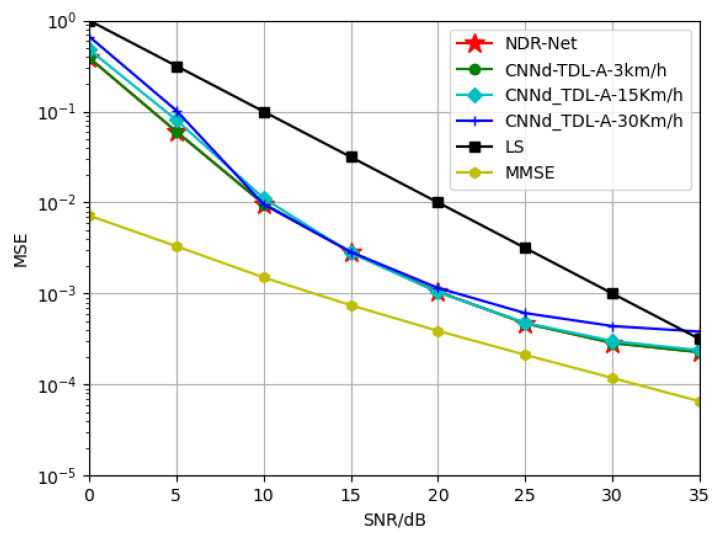
Generalization performance of TDL-A channels at different training velocities when testing velocity = 30 Km/h.

**Figure 7:**
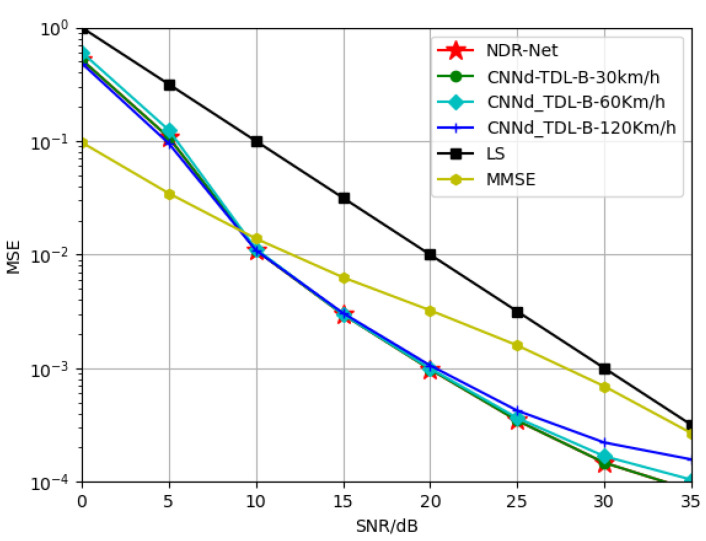
Generalization performance of TDL-B channels at different training velocities when testing velocity = 120 Km/h.

In this part, in order to explore the generalization performance of the proposed algorithm NDR-Net on the moving speed under the same channel model, SNR is 15 dB, TDL-A channel is 3 Km/h and 30 Km/h respectively, and TDL-B channel is 30 Km/h and 120 Km/h respectively. The TDL-C channel is trained at 120 Km/h and 350 Km/h respectively. The Doppler shifts corresponding to 3 Km/h, 30 Km/h, 120 Km/h and 350 Km/h are 9.72 Hz, 97.22 Hz, 388.89 Hz and 1134.26 Hz respectively. Figure 6 and Figure 7 show the simulation results for the test channels TDL-A-30km/h and TDL-B-120Km/h, respectively. It can be seen that in the same channel, the algorithm is not sensitive to the moving speed. When the test SNR is also 15 dB, the model also has good adaptability when the moving speed is not matched. Even under low or high SNR, the proposed algorithm can accurately estimate the doppler shift and determine the correct moving speed. In other words, even if SNR and moving speed do not match, the DL algorithm can achieve better estimation effect than the traditional algorithm and has good robustness.

**Figure 8:**
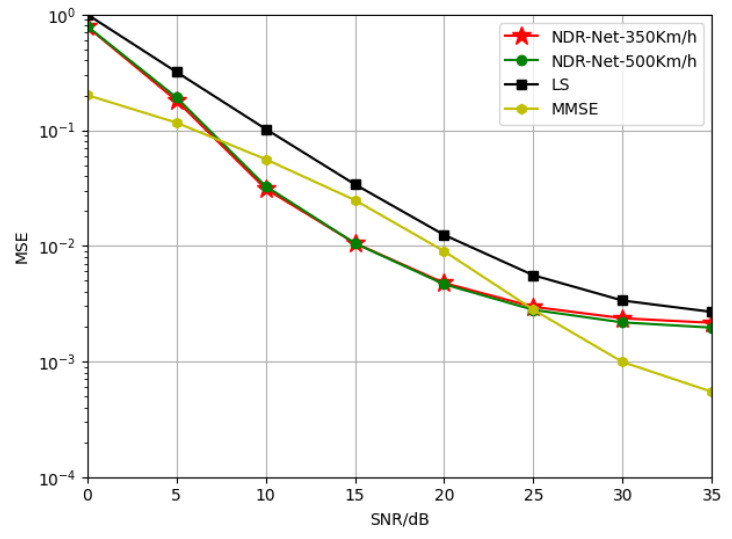
Generalization performance of NDR-Net when the training channels are TDL-C-350Km/h and TDL-C-500Km/h and the test channel is TDL-C-500Km/h.

The maximum mobile speed supported by 5G communications has reached 500 Km/h. Figure 8 represents the generalization effect under the TDL-C channel model trained at 350 Km/h and 500 Km/h moving speed respectively. It can be seen that the SNR loss of the model trained at 500 Km/h is no more than 0.1 dB compared with that of the model trained at 350 Km/h, indicating that the proposed algorithm is not sensitive to the moving speed. Thus the proposed algorithm has an excellent generalization effect even when the velocity is very large.

#### 5.2.3. Generalization of NDR-Net on Channel Model

In this part, In order to explore the generalization performance of the proposed algorithm NDR-Net on the channel model, $CNN_{N}$ is used to estimate the RMS delay spread value of the channel. Similar to the idea proposed in Section 5.2.1, $CNN_{D}$ is trained on three channel models, namely TDL-A, TDL-B and TDL-C, to obtain three network models. Then, according to the root mean square delay spread value, the corresponding trained denoising network is selected for denoising, so as to achieve the purpose of adaptive channel model selection. Among them, the RMS delay spreads of the three channels are 10 ns, 100 ns and 1000 ns, respectively. Because the number is too small, in order to facilitate the model to compare the labels, they are scaled and multiplied by $10^{7}$, so that the labels of $CNN_{N}$ become 0.1 s, 1 s and 10 s.

**Figure 9:**
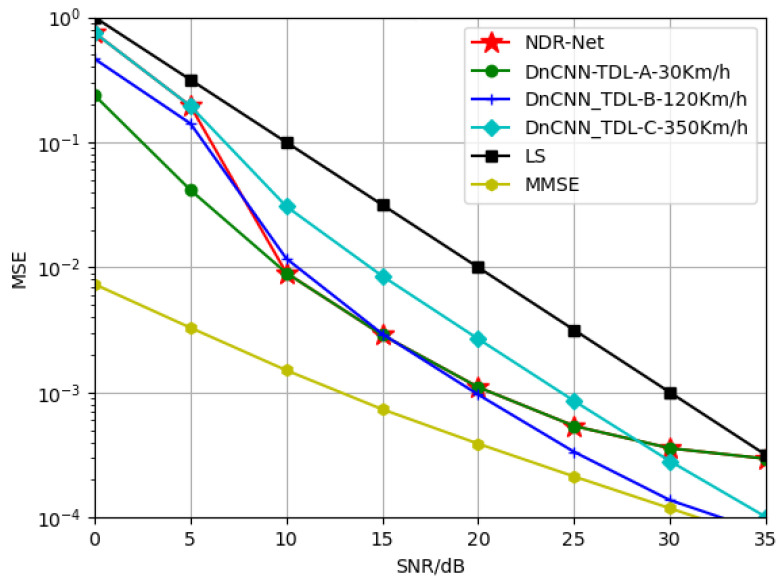
Generalization performance of different channel estimators when the training SNR = 15 dB and the testing channel is TDL-A-30Km/h.

Figure 9 shows that when the fixed training SNR is 15 dB, the model is trained under TDL-A-30Km/h, TDL-B-120Km/h and TDL-C-350Km/h, and tested under TDL-A-30Km/h. The channel model generalization performance of different channel estimation algorithms obtained is compared. It should be noted in particular here that the proposed algorithm is not sensitive to the Doppler shift due to velocity, since it has been shown in the previous subsection. This means that the effect of velocity on the algorithm is small and almost negligible. Thus the presence of velocity has no effect on the results of the experiment. As can be seen from the simulation results, in the case of low SNR, due to the large noise component, the error between the RMS delay extension of the TDL-B channel estimated by $CNN_{N}$ network and the real value is large, resulting in the channel model is easy to be judged as the mismatched channel model TDL-C, resulting in misjudgment in the selection of the model. Under high SNR, because SNR and channel model do not match at the same time, the model trained under TDL-B and TDL-C channel can be well adapted to the test channel.

**Figure 10:**
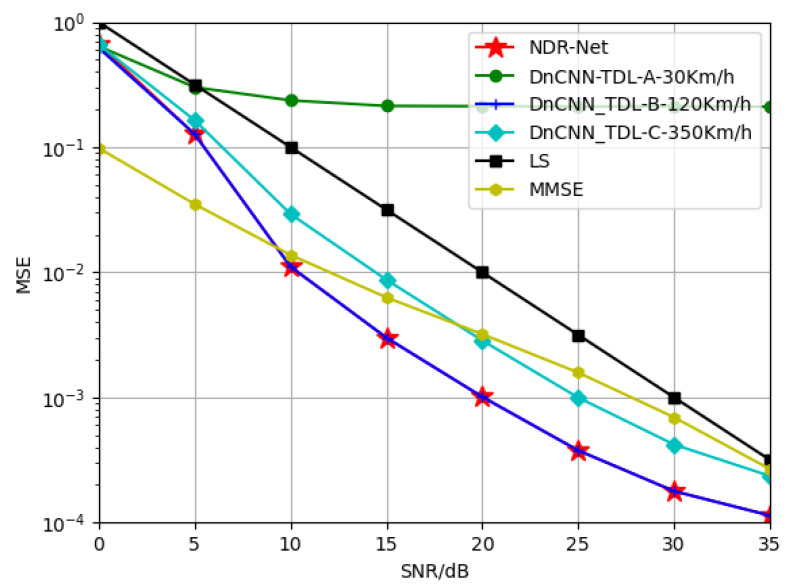
Generalization performance of different channel estimators when the training SNR = 15 dB and the testing channel is TDL-B-120Km/h.

**Figure 11:**
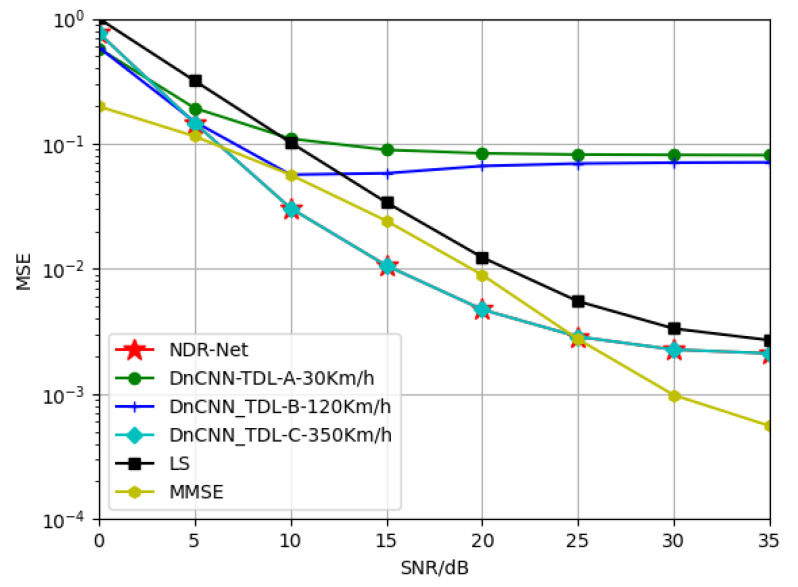
Generalization performance of different channel estimators when the training SNR = 15 dB and the testing channel is TDL-C-350Km/h.

In Figure 10, the test channel model is changed to TDL-B-120Km/h. At this time, the error of TDL-A channel is extremely large and can no longer meet the requirements. Similarly, the test channel model is changed to TDL-C-350Km/h in Figure 11. At this time, TDL-A and TDL-B cannot satisfy the requirements due to too large errors. The MMSE algorithm always has the best performance at low SNR since it takes into account the noise factor. At high SNR, NDR-Net performs better than LS estimates, but MMSE also outperforms NDR-Net when the channel is the more complex TDL-C.

#### 5.2.4. Generalization Ability of NDR-Net

A. Joint estimation of SNR, Channel model and velocity

Since the fixed training SNR is 15 dB when exploring the generalization of NDR-Net to the channel model and moving speed, it is easy to misjudge the channel model under low SNR. Meanwhile, the performance of the training model under the harsh environment of high SNR is better than that of the channel model matching the test channel. Therefore, in order for NDR-Net to improve channel selection capability under low SNR and model matching degree under high SNR, it is necessary to conduct joint estimation of SNR and channel model.

**Figure 12:**
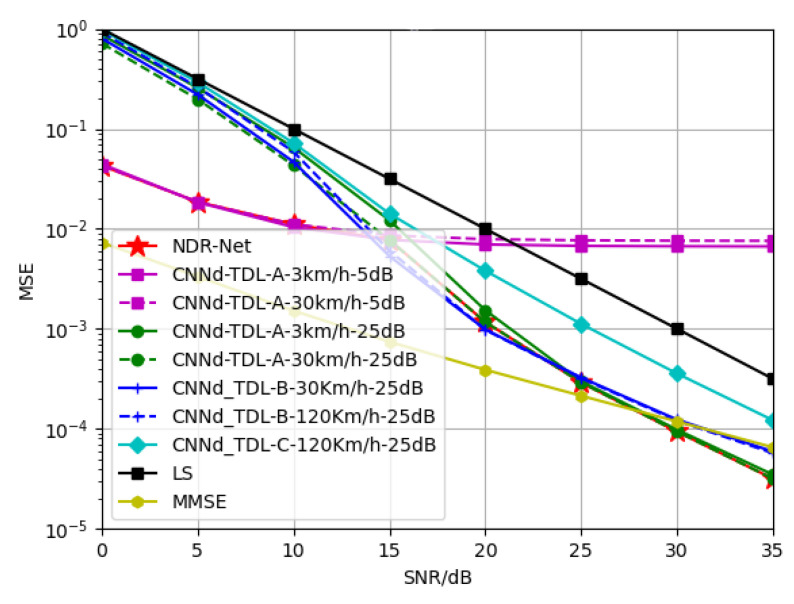
Generalization performance of different channel estimators when the test channel is TDL-A, SNR = 15 dB, Velocity = 30 Km/h.

**Figure 13:**
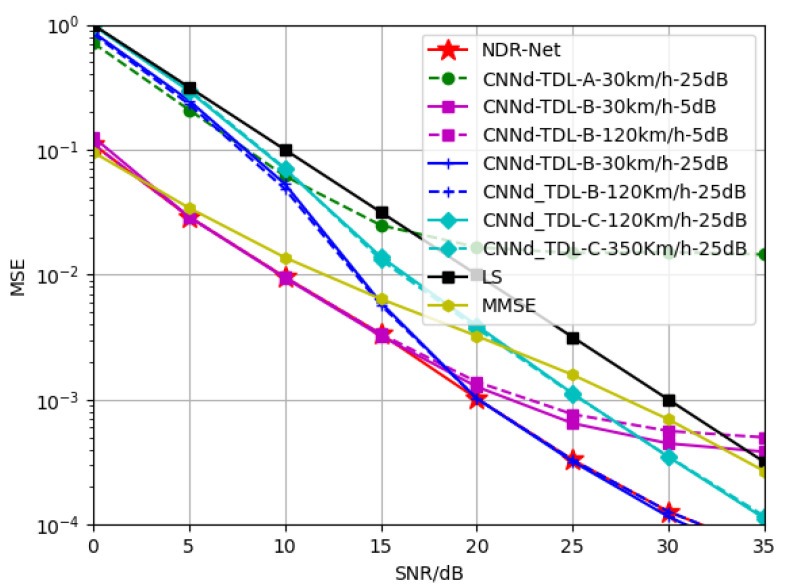
Generalization performance of different channel estimators when the test channel is TDL-B, SNR = 15 dB, Velocity = 120 Km/h.

**Figure 14:**
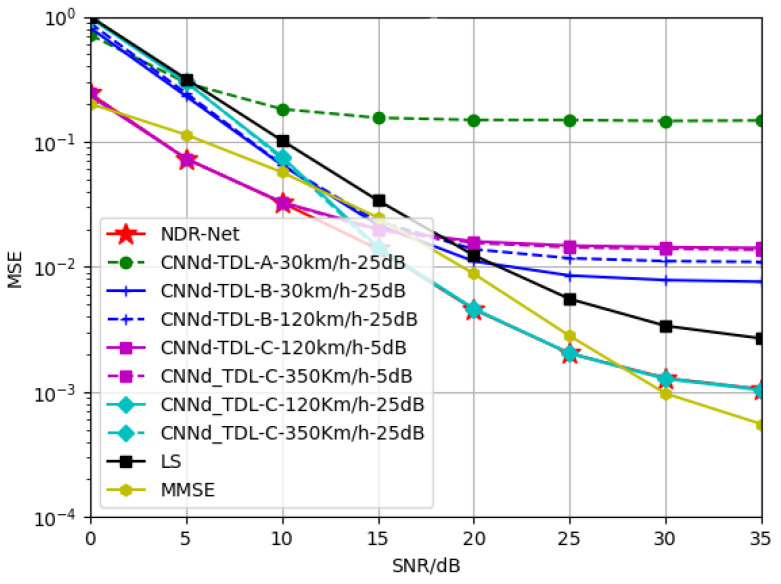
Generalization performance of different channel estimators when the test channel is TDL-C, SNR = 15 dB, Velocity = 350 Km/h.

Therefore, the model trains the TDL-A, TDL-B and TDL-C channels respectively with SNR of 5 dB and 25 dB. At this time, the corresponding moving speed of different channels is taken into account, and the environmental factors are extended. Figure 12, Figure 13 and Figure 14 show the simulation results of the proposed algorithm when SNR is 15 dB and the channel model is TDL-A-30Km/h, TDL-B-120Km/h and TDL-C-350Km/h respectively. It can be seen that since both SNR and channel model are taken into account in NDR-Net, the channel model corresponding to the test environment can always be selected by NDR-Net when testing under 0–35 dB. Since the TDL-A channel has the smallest number of paths, the MMSE can take advantage of the noise information in the channel and therefore outperforms the NDR-Net in terms of low SNR. when the channels are TDL-B and TDL-C, the MMSE estimated performance is limited at this point. It can be seen that when the MSE is $10^{-2}$, the loss of SNR performance is 10 dB for the test at TDL-A-30Km/h, the SNR performance improves by 2 dB at TDL-B-120Km/h, and the SNR performance improves by about 3 dB at TDL-C-350Km/h.

B. Generalization of NDR-Net under other factors

Because of the complexity and variability of the actual high-speed mobile environment, it is not sufficient to consider only SNR, channel model and mobile speed. In OFDM systems, in addition to the Doppler shift caused by the moving speed, there is often a Carrier Frequency Offset (CFO) between the transmitter carrier frequency and the receiver local oscillator. This offset may cause a rotation in the phase of the signal and ICI, resulting in an error in the received signal. On the other hand, the OFDM signal needs to sum all subcarriers after IFFT operation, so the transmit signal in the time domain will have high peaks, resulting in an OFDM system with a high PAPR. High PAPR can lead to in-band distortion, which can reduce system performance. To reduce the PAPR, the signal can be clipped after it has been modulated.

**Figure 15:**
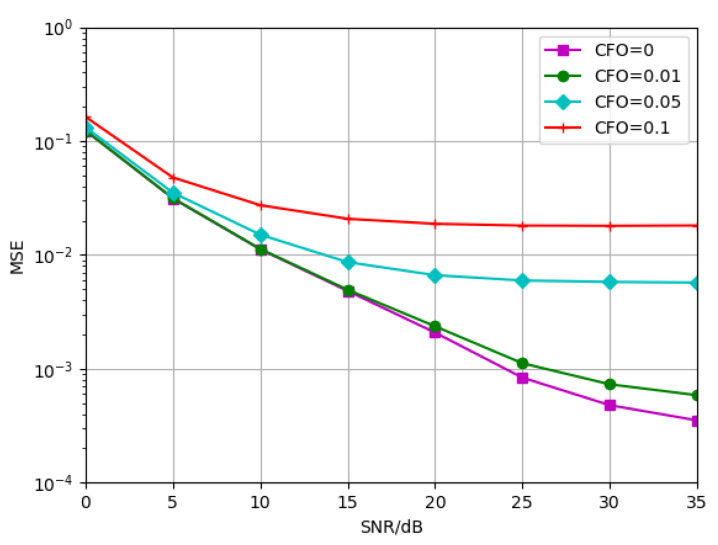
Generalization performance of NDR-Net trained at CFO = 0 and tested under the conditions of CFO = 0, 0.01, 0.05, 0.1, respectively.

Therefore, to further verify the effectiveness of the proposed algorithm, we first consider the situation that there is a deviation of the principal vibration frequency in the actual environment. The proposed algorithm is trained at TDL-B-120Km/h with CFO = 0. The tests were conducted at the normalized CFO of 0, 0.01, 0.05 and 0.1, respectively. The simulation results are shown in Figure 15. It can be seen that the proposed algorithm is very sensitive to the deviation of CFO. When the normalized CFO is in the range of $\left[0,0.01\right]$, the effect on the signal is small and only a small angle of phase rotation is produced. When the CFO is 0.01, the estimated performance of the model is not affected at low SNR, and the performance loss is gradually enlarged at high SNR. It can be seen that the SNR loss is 1 dB at an MSE of $10^{-3}$. Model performance is limited at high SNR for CFO is 0.05 or above. This is because the model already appears flat after 20 dB. To mitigate the impact of CFO on the channel estimation accuracy, the CFO needs to be compensated at the receiver side. This is what we need to study in the future.

**Figure 16:**
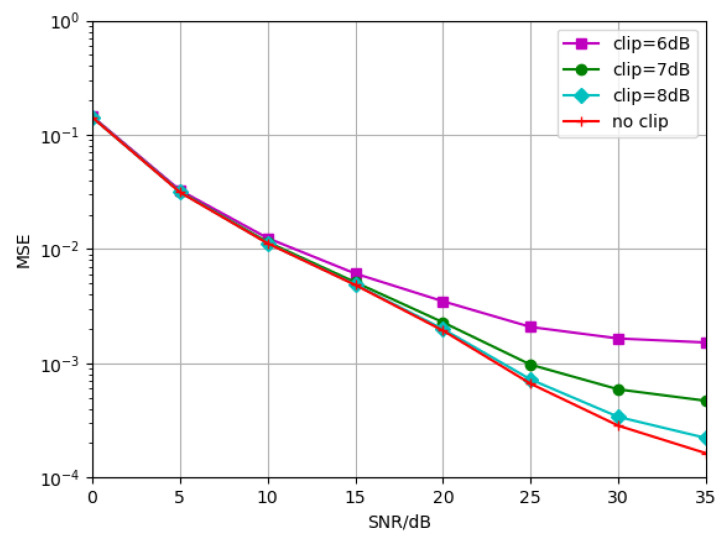
Generalization performance of NDR-Net trained without signal clipping, tested under the conditions of signal without clipping, signal clipped to 6 dB, 7 dB and 8 dB, respectively.

We also consider the case where the PAPR of the transmitted OFDM signal is high. To reduce the impact of high PAPR, the signal is clipped before it passes through the channel. Figure 16 represents the proposed algorithm trained at TDL-B-120Km/h without signal clipping. The simulations are performed under the conditions of signal without clipping, signal clipped to 6 dB, 7 dB and 8 dB, respectively. Firstly, as can be seen from the simulation results, when the modulated signal is clipped to 6 dB, its MSE curve appears flat when the SNR is greater than 30 dB, so the SNR loss is gradually enlarged compared with the estimated performance tested under unclipping. Secondly, there is almost no SNR performance loss when the MSE is $10^{-2}$. For example, when the MSE is 0.019, the SNR performance loss is about 5 dB. Therefore, in general, the proposed algorithm is robust to PAPR.

## 6. Conclusions

In this paper, a cascaded network NDR-Net is proposed to address the problem of insufficient estimation performance of traditional channel estimation algorithms and existing DL-based channel estimation algorithms in OFDM systems. This model can simultaneously solve the problems of signal-to-noise ratio, channel model and mobile speed mismatch in 5G multipath time-varying channels.

The proposed NDR-Net is compared with LS, MMSE estimation algorithm and a single denoising model under different SNR, three 5G channels and mobile speeds by considering the signal as full pilot, using QPSK modulation scheme and MSE as evaluation function. Simulation results show that the proposed model can be applied to standard 5G channels, and it also improves by nearly 5–7 dB compared with the MMSE algorithm in the case of SNR mismatch, and can overcome the problem of too small noise adaptation interval encountered by the single denoising model. When the moving velocities in the same scene are not matched, the proposed model can always be applied to different Doppler shifts and has certain robustness. When the Channel model is not matched, the proposed algorithm can always get the optimal result by jointly estimating the SNR and Channel model, which can solve the problem of both SNR and Channel model mismatch. In addition, we also verify the generalization performance of the proposed algorithm in the presence of local oscillation offset and signal clipping at both transceivers. The results demonstrate that the proposed algorithm performs well under the condition that the local oscillation offset is less than 0.01 dB and is robust to the channel estimation after signal clipping.

For simplicity, the overall system is supposed to be time-synchronized. The proposed algorithm focuses on typical scenarios of 5G terrestrial communication, including pedestrian, vehicle and urban channels, which to a large extent can meet the basic communication needs of most 5G scenarios. Notice that, the practical 5G communication environment covers more complex scenarios, the cost and overhead will increase dramatically if more channel models are trained separately. Therefore, the proposed algorithm is suitable for scenarios that require less real-time computing and have more abundant computing resources. In future work, deeper research and analysis will be conducted from the perspectives of how to reduce the complexity of the model, how to reduce the noise introduced by pilot and channel estimation under high-order modulation.

## Figures and Tables

**Table 1:** List of related works.

Reference	Research Direction	Contribution	Limitation
[16]	Improving the channel estimation accuracy of OFDM systems in high-speed mobile scenarios	Proposed ChanEstNet, which can avoid the fast time-varying and non-smooth characteristics in high-speed moving scenes that lead to the limited estimation performance	Only the two extreme speeds under the same channel are considered
[17]	Consider the effect of ICI to improve the accuracy of channel estimation under fast time-varying channels	For fast time-varying channels, a novel network ICINet for sensing ICI is proposed	The number of carriers causing ICI cannot be found according to the actual situation
[19]	Real-time training data collection and online channel estimation for OFDM systems	Proposed a new training data generation scheme and an LML-based online channel estimation method, which can adapt to real-time channel changes	The proposed generation scheme can provide limited data
[22]	Simplify network structure and reduce training overhead of ML-based estimators for channel estimation in large-scale MIMO systems	A KDML-based channel denoising model is designed and the network structure is simplified by using domain knowledge	Limited to a small range of noise levels
[23]	Treat the fading channel as a two-dimensional image, and simulate channel interpolation and denoising with two networks respectively	Proposed ChannelNet, which outperforms the estimated MMSE method for a specific SNR value	The network must be retrained for each SNR value
[24]	Improved denoising performance for real noise images in mmWave Massive MIMO systems	Proposed CBDNet, the asymmetric learning can adjust the estimated noise level map and interactively reduce the noise in the channel matrix	No consideration of the actual channel model and movement speed mismatch with training phase
Proposed	Improving the generalizability of channel estimation algorithms under environmental mismatch for OFDM systems	Proposed NDR-Net that can be used for channel estimation under unknown noise levels	Restricted to a limited variety of channel models

**Table 2:** Noise level estimates for two pooling methods.

Method	Noise Level (SNR/dB)							
	0	5	10	15	20	25	30	35
Maxpooling	0	4.83	9.86	14.76	19.37	24.06	28.36	33.34
Averagepooling	0	5.13	10.13	15.22	20.14	24.76	29.49	34.85

**Table 3:** RMSE for two pooling methods.

Method	RMSE							
	0	5	10	15	20	25	30	35
Maxpooling	0	0.18	0.15	0.25	0.64	0.95	1.64	1.67
Averagepooling	0	0.16	0.15	0.23	0.17	0.26	0.54	1.19

**Table 4:** The computational complexity of different DL networks.

Methods	Number of Parameters	Number of FLOPs
NDR-Net	1231K	67K
KDML [22]	112K	60K
ChannelNet [23]	678K	358K
CBDNet [24]	6792K	166k

**Table 5:** Experimental Settings for OFDM signal generation.

Parameter	Value
Carrier frequency	3.5 GHz
System bandwidth	20 MHz
Sample rate	30.72 MHz
Subcarrier interval	15 KHz
Number of valid subcarriers	1200
Number of Resource Block	100
FFT points	2048
The length of time slot	0.5 ms
OFDM symbols per time slot	7
Number of OFDM symbols	14
The length of the CP	Normal CP
SNR range	0 dB to 35 dB
Modulation scheme	QPSK
Channel model	TDL-A, TDL-B, TDL-C

**Table 6:** Experimental Settings in NDR-Net model.

Parameter	Value
Train set size	12,000
Validation set size	4000
Test set size	4000
Num layer	15
Learning Rate	0.001
Batch Size	8
Num Epochs	2000
Optimizer	Adam

## Data Availability

Not applicable.
